# Supplementary material for: Inspiratory response and side-effects to rapid bilateral magnetic phrenic nerve stimulation using differently shaped coils: implications for stimulation-assisted mechanical ventilation
Source: Respir Res. 2022 Dec 17;23:357. doi: 10.1186/s12931-022-02251-y (PMC9758474; doi:10.1186/s12931-022-02251-y)
Supplement: Supplementary file 3 — Additional file 3. Correlations between tidal volumes in response to single-train rapid bilateral anterior magnetic phrenic nerve stimulation and anthropometric variables. [file 12931_2022_2251_MOESM3_ESM.pdf]

Additional file 3. Correlations between tidal volumes in response to single-train rapid bilateral anterior magnetic phrenic nerve stimulation and anthropometric variables.

	V_T and BMI		V_T and body fat percentage		V_T and neck circumference		V_T and neck fat percentage	
**Coil pair**	r	*P-value*	r	*P-value*	r	*P-value*	r	*P-value*
**Parabolic**								
15Hz, 20%	-0.02	0.945	0.46	0.160	-0.06	0.868	0.31	0.355
15Hz, 30%	0.00	0.993	**0.65**	**0.030**	-0.22	0.510	0.45	0.162
20Hz, 20%	0.02	0.944	0.44	0.175	-0.05	0.892	0.31	0.360
20Hz, 30%	**0.69**	**0.041**	0.52	0.187	0.34	0.405	**0.75**	**0.033**
25Hz, 20%	0.02	0.947	0.49	0.129	0.01	0.973	0.39	0.231
25Hz, 30%	0.39	0.346	0.66	0.109	-0.02	0.967	0.71	0.074
30Hz, 20%	0.04	0.912	0.53	0.094	0.01	0.984	0.43	0.184
30Hz, 30%	0.30	0.560	0.87	0.058	-0.24	0.698	0.72	0.169
Maximal responses	-0.01	0.971	**0.68**	**0.023**	-0.25	0.461	0.53	0.091
**D-shape**								
15Hz, 20%	-0.14	0.675	-0.09	0.792	0.42	0.204	0.09	0.792
15Hz, 30%	0.17	0.607	0.18	0.604	0.42	0.199	0.30	0.370
20Hz, 20%	-0.09	0.771	-0.20	0.548	0.56	0.075	0.07	0.828
20Hz, 30%	0.46	0.152	-0.10	0.780	**0.68**	**0.032**	0.24	0.503
25Hz, 20%	0.06	0.863	0.11	0.749	0.44	0.171	0.29	0.386
25Hz, 30%	**0.65**	**0.041**	0.23	0.546	0.48	0.192	0.53	0.143
30Hz, 20%	-0.10	0.753	-0.01	0.966	0.40	0.227	0.20	0.556
30Hz, 30%	0.61	0.063	-0.17	0.653	**0.73**	**0.025**	0.21	0.593
Maximal responses	0.08	0.809	-0.05	0.878	0.40	0.228	0.13	0.708
**Butterfly**								
15Hz, 20%	-0.24	0.485	-0.38	0.279	0.34	0.330	-0.23	0.519
15Hz, 30%	-0.21	0.540	0.07	0.846	0.20	0.582	0.06	0.865
20Hz, 20%	-0.41	0.205	-0.38	0.284	0.32	0.362	-0.27	0.442
20Hz, 30%	-0.35	0.288	0.01	0.986	0.11	0.764	-0.01	0.971
25Hz, 20%	-0.35	0.291	-0.33	0.354	0.35	0.330	-0.20	0.575
25Hz, 30%	-0.42	0.231	0.02	0.967	0.13	0.730	-0.03	0.933
30Hz, 20%	-0.49	0.125	-0.26	0.469	0.14	0.703	-0.19	0.591
30Hz, 30%	-0.38	0.255	-0.09	0.812	0.14	0.701	-0.02	0.963
Maximal responses	-0.02	0.962	0.36	0.311	-0.01	0.970	0.34	0.330

Definition of abbreviations: V_T = tidal volume; BMI = body mass index; r = Pearson correlation coefficient; Hz = hertz. Significant correlations are displayed as bold text.
